# Predictors of Stress of Parents of a Child with Cancer: A Jordanian Perspective

**DOI:** 10.5539/gjhs.v5n6p81

**Published:** 2013-09-04

**Authors:** Rami Masa’Deh, Jacqueline Collier, Carol Hall, Fadwa Alhalaiqa

**Affiliations:** 1 School of Nursing, Applied Science Private University, Amman, Jordan; 2 School of Allied Health Professions, Queen’s Building, University of East Anglia, Norwich, UK; 3 School of Nursing, Midwifery & Physiotherapy, Queen’s Medical Centre, University of Nottingham, Nottingham, UK; 4 School of Nursing, Philadelphia University, Amman, Jordan

**Keywords:** parent, child, cancer, stress

## Abstract

**Background:**

Most paediatric oncology studies agree that being parents of a child with cancer is an emotionally stressful event. Although an increasing number of studies have investigated psychological stress of parents of a child with cancer, few of these studies have included both parents or investigated the predictors of high stress levels for the mothers and the fathers. Moreover, studies published over the last few decades were limited to Western countries and have shown inconsistent findings about parental perceived stress whose children have cancer. This study explored differences in predictors of perceived stress between Jordanian mothers and fathers of children with cancer.

**Methods:**

This study involved a survey of 300 couples parenting a child with cancer. Participants answered the Arabic version of the Perceived Stress Scale 10-items, demographic and characteristics check list questionnaires. The main aims were to measure perceived stress levels for mothers and fathers, explore the predictors associated with high perceived stress levels and make a comparison between them.

**Findings:**

Mothers reported significantly higher stress levels than fathers (p<0.001), with a large effect size (0.30). Some of the factors associated with mothers and fathers high stress levels affected both parents whereas employment status affected only fathers’ stress levels.

**Conclusions:**

These findings indicate the need to work with the mothers and the fathers with a child with cancer in Jordan to recognise their psychological needs at the time of diagnosis and followed by on-going psychological support for both parents.

## 1. Introduction

Chronic and acute illnesses still affect children all over the world. Childhood chronic medical conditions pose significant stressors to parent of children with these conditions, and the parents of children with cancer are no exception (Dixon-Woods, Young, & Heney, 2005; Warner et al., 2011).

Parents have been found to be psychologically affected by their child’s diagnosis, treatment, side-effects of the treatment and child’s health status (Jurbergs, Long, Ticona, & Phipps, 2009; Maurice-Stam, Grootenhuis, Brons, Caron, & Last, 2006). Previous studies have shown that parents may live with continuous uncertainty about the outcomes (Eiser & Upton, 2007; P. Sloper, 1996) and may have to live with the threat of relapse or death for years (Bjork, Wiebe, & Hallstrom, 2005). They often need to change their family daily routine and some of their roles and responsibilities (Patterson, Holm, & Gurney, 2004; Rajajee, Ezhilarasi, & Indumathi, 2007; P. Sloper, 1996). Moreover, parents have reported additional burdens; for example, issues in their employment and financial status (Dockerty, Skegg, & Williams, 2003; Gravestock, McDowell, & Vale, 2011; Limburg, Shaw, & McBride, 2008; Miedema, Easley, Fortin, Hamilton, & Mathews, 2008), burden in their family relationships (Bjork et al., 2005) and issues in caring for other children (Rajajee et al., 2007).

The above stressors are reported to affect both mothers and fathers parenting children with cancer. However, it is important to note that mothers and fathers have been found to have different levels of perceived stress (Norberg, Lindblad, & Boman, 2006; Yeh, 2002). Many of the studies have found that the parent’s gender is a risk factor for high perceived stress level and therefore some psychological problems (Bayat, Erdem, & Gul Kuzucu, 2008; Best, Streisand, Catania, & Kazak, 2001; Kazak, Boeving, Alderfer, Hwang, & Reilly, 2005; Pöder, Ljungman, & Essen, 2008). For example, mothers of a child with cancer reported higher levels of anxiety, depression and sadness than fathers (Bayat et al., 2008; Norberg et al., 2006). Mothers also have been found to be at higher risk of post-traumatic stress disorder than fathers (Best et al., 2001; Kazak et al., 2005; Pöder et al., 2008). However, these differences may be a reflection of the general finding that women report poorer psychological well-being than men (Ptacek, Smith, & Dodge, 1994). Monnier et al. (1998) suggested that this may be because women are more willing to report symptoms than men as expressing stress is considered to be more socially acceptable for them than for men, bringing a cultural dimension to the consideration of stress perception. In particular, many researchers report that the cultural background of the parents has an effect on their stress levels when caring for a child with cancer (Bozo, Anahar, Ates, & Etel, 2010; Han, 2003; Johns et al., 2009; Patistea, Makrodimitri, & Panteli, 2000; Rajajee et al., 2007; Wong & Chan, 2006). Thus, exploring both of the parents’ experiences in the context of children with cancer in various cultures is important.

Tayeb et al. (2010) suggested that marital relationships, parental gender roles and responsibilities differ across cultures. Many people in Jordan report rather unanimously, across both genders and age groups, that husbands/fathers should provide the family income and be responsible for financial decisions while wives/mothers should have the prime responsibility for housework and care for their children (Jordanian Higher Council for Youth, 2009). This may have an impact on how Jordanian mothers and fathers react when having a child with cancer. Additionally, many trans-cultural nursing theorists and researchers believe that there is an effect of the surrounding environment and cultural values on human beings (Giger & Davidhizar, 2008; Leininger & McFarland, 2006; Sagar, 2012).

It is acknowledged that people differ in their reactions to stressful events and this may depend on many factors such as their previous experiences and differences in their roles, responsibilities and coping mechanisms. Moreover, religion, culture and the nature of marital relationship between couples are very important factors affecting how parents of children with cancer respond to stress. However, understanding parental stress of mothers and fathers of a child with cancer in an Arab Muslim community is still not clear and has not been a focus of research. Therefore, exploring perceived stress in mothers and fathers of a child with cancer in such a community will enable healthcare providers to understand the parental stressors, needs and the differences between those parents in how they deal with stress as a result of their child’s illness. Thus, culturally relevant care can be offered for both mothers and fathers taking into consideration the effects of culture, religion and social contexts. Approximately 200 new cases of childhood cancer are diagnosed in Jordan each year (Jordan National Cancer Registry, 2008). Due to improvements in childhood cancer treatment, the overall survival rate of all childhood cancers combined has improved. This is evident in the United States and also in Jordan where the focus of this study is to be considered (American National Cancer Institute, 2012; Jordan National Cancer Registry, 2008). Nevertheless, within the paediatric oncology literature there is agreement that being parents of a child with cancer is still an emotionally stressful event (Bjork et al., 2005; Kazak et al., 2005; Lindahl Norberg & Boman, 2008; Maurice-Stam et al., 2006; Maurice-Stam, Oort, Last, & Grootenhuis, 2008; McCarthy et al., 2009; Pai et al., 2007; Pöder et al., 2008; Svavarsdottir, 2005; Witt et al., 2010). However, little is known about the stressors of parenting a child with cancer in Jordan.

The purpose of the study were to measure the perceived stress levels of the participants; make comparisons between mothers and fathers caring for a child with cancer in terms of their perceived stress levels; and the need to predict the factors that lead to differences in perceived stress levels in parents who are caring for a child with cancer.

Hypotheses: (a) mean levels of perceived stress score would be higher for mothers than fathers; (b) mothers’ and fathers’ levels of stress would be positively correlated; and (c) mothers will report different predictors to stress from the fathers.

## 2. Methods

The stress and coping model identified by Lazarus and Folkman (1984) suggests that stress may result from an imbalance between individual parental stressors and their resources to buffer it. Accordingly, there is a need to investigate both the mothers’ and the fathers’ experiences when having a child with cancer. This cross-sectional survey was carried out in Jordan using an Arabic version of the Perceived Stress Scale 10-items (PSS10) and characteristics check list. The questionnaire was completed by 300 couples with a child who has cancer.

### 2.1 Ethical Considerations

Ethical approval was gained from the Jordanian Ministry of Health, the Jordan Royal Medical Services, University of Nottingham and the Cancer Centre involved in this study and permission to proceed with the study was secured. Participants’ informed consent was gained; voluntary participation and confidentiality were guaranteed.

The participants were advised (both verbally and in writing) in every stage of the research about the nature of the voluntary participation and their right to withdraw at any stage and any time without giving a reason, causing no penalty or loss of benefits to them or their ill children.

A study number was given to each participant in a way that enabled the researcher to link couples together. Moreover, questionnaire data have been stored in a secure place and were only available to the researchers and not used by anyone else without the consent of the participants. The participants were given full information about the purpose of the data they will present and that the questionnaires will be destroyed seven years after the completion of the study.

### 2.2 Participants

As per ethical approval, parents of a child with cancer were approached from six hospitals that are known to treat childhood cancer, represent all health sectors in Jordan and are located in the three main Jordanian cities (Amman, Irbid and Zarqa). These cities cover more than 75% of the Jordanian population (Jordanian Department of Statistics, 2008). Statistics have shown that these hospitals treat more than 85% of the childhood cancer patients in the country (Jordan National Cancer Registry, 2007).

The population of this study was mothers and fathers (couples) known to have a child diagnosed with any type of cancer in Jordan.

Parents who met the following criteria were eligible to participate in the study:

a) Parents with a child diagnosed with any type of cancer and attending for his/her treatment either in a paediatric oncology ward or attending follow-up paediatric oncology clinics in any of the six selected hospitals; and
b) Parents should be able to speak, understand, read and write Arabic.

Parents were excluded if they:

a) Have hidden the medical diagnosis from their ill child and/or siblings and/or relatives and friends (those parents might have different stressors affecting them and different ways of social support); and/or
b) Were parents of a dying child or with a life expectancy of only few weeks.

### 2.3 Sampling

All those who attended any of the selected hospitals during the data collection period of this study were approached. Using non-random sampling allowed the researcher to find parents of a child with cancer who reflect the wider parental population at varying stages of treatment as per the aims of the research question and proposed forms of data analysis.

In this study, firstly, one goal of the proposed study was to test the null hypothesis that the stress levels of the two population (the mothers’ and the fathers’ means) are equal. The criterion for significance (alpha) was set at 0.05, 2-tailed, so that an effect in either direction will be interpreted.

A sample size calculation was carried out using Sample Power software. A previous study conducted in Jordan had used the Perceived Stress Scale of ten items (PSS10) to illustrate perceived stress levels for university students. This study found that the females mean score was 22.7 (standard deviation 7.7) whereas males mean scores was 21.0 (standard deviation 7.6) (Hamdan-Mansour & Dawani, 2008). Assuming that the mean difference is 1.7 (corresponding to means of 22.7 versus 21.0) and the common within-group standard deviation is 7.7 (based on standard deviation estimates of 7.7 and 7.6) as in the previous Jordanian study by Hamdan-Mansour and Dawani (2008), then a sample size of 319 and 319 for the two groups will have power of 80.0% to yield a statistically significant result. Accordingly, the proposed sample size is enough to detect a 1.7 difference in the mean stress score between mothers and fathers of a child with cancer in Jordan.

### 2.4 Data Collection Procedure

#### 2.4.1 Research Team

Two research nurses in each selected hospital were assigned. They approached the participants, handed out the information sheets, received the consent from parents who wanted to participate in the study, handed out the questionnaires and answered participants’ questions about the nature of the study. The nurses were trained in conducting research, authorized to carry out such kind of research, assigned by the Jordanian health ethical committee to work in this study and had participated in different research in the past. The researcher clarified and explained each point about the project to the research nurses. The researcher and the nurses agreed on a recruitment script in order to recruit in an identical manner. Meetings were held between the researcher and the assigned research nurses regularly every three days at each location. The researcher contacted those nurses everyday either at the location or by telephone. The researcher’s contact details were available, so the participants and the assigned nurses had access to the researcher and they could contact the researcher for any enquiry.

#### 2.4.2 Gaining Access to Participants

After gaining ethical approval, the researcher placed explanatory posters on notice boards within the paediatric departments and outpatient clinics in all selected hospitals describing the study to ensure that parents had an idea of what is involved in the research before nurses talked to them individually. The identical recruitment script used by all research nurses and the explanatory posters in all selected settings helped to reduce any recruiting bias. One of the research nurses in each hospital approached the parents in the paediatric ward or in the clinic and discussed the information sheet with them. The research nurses used a recruitment log and recorded who was approached, consented, did not reply and any other notes.

The participants were asked to call either of the two research nurses or the researcher within a week after having been given the information sheet. The research nurses’ and researcher’s contact details and phone numbers were clearly outlined in the information sheet. Parents could call then for any enquiries about the study and those who wanted to participate could arrange with any of the research nurses on a time and place to sign the informed consent. By signing the consent, the participant accepted to willingly take part in the questionnaires and the demographic and characteristics checklist which were handed out in a separate envelope to each parent. Participants were provided with a stamped addressed envelope.

### 2.5 Measures

#### 2.5.1 The Arabic Version of Perceived Stress Scale 10-Items Questionnaire (PSS10)

It has been identified that stress occurs when perception of the environmental demands exceeds the perceived resources (Lazarus, 2006). The questions used in the PSS10 questionnaire are all about demands and resources. This reflects the theoretical framework underpinning this study. The questions measure the participant’s feelings as to whether they have enough resources in comparison with the demands (Cohen, Kamarck, & Mermelstein, 1983). The questions are set to be quite simple and general in their nature and are judged over a broad spectrum in order to relate to participants with varying social circumstances (such as parents of a child with cancer) (Cohen et al., 1983). Due to the fact that this questionnaire is short and easy to complete, parents of a child with cancer were participated have little difficulties or burden in participation. This is relevant to this investigation with its focus on personal perceived stress of parents caring for a child with cancer in Jordan. Moreover, the PSS10 has been used in Arabic language in several studies (Al-Hassan & Wierenga, 2000; Hamdan-Mansour & Dawani, 2008; Hattar-Pollara & Dawani, 2006) and tested in Jordan with reliability of 0.68 (Hamdan-Mansour & Dawani, 2008).

The PSS10 is a rating scale which can be completed in 4 minutes (Cohen et al., 1983). The total score is obtained by reversing the scores of the four positive items (4, 5, 7, 8), e.g. 0=4, 1=3, 2=2, etc. (e.g. item number 8, in the last month, how often have you felt that you were on top of things?). The other items have been written in a negative form (e.g. in the last month, how often have you found that you could not cope with all the things that you had to do?). Item responses range from never (0) to very often (4). The higher the score means the higher stress level.

#### 2.5.2 Demographic Data and Characteristics Check List

A participant characteristics check list included the following information: participant’s gender, the primary caregiver of the ill child, the decision maker in the family in regards to the ill child, participant’s education level, participant’s employment status, age of the participant, source of the medical information, help from others and feeling stress. Also, perception of family finances, distance from the hospital, age of the ill child, sex of the ill child, date of diagnosis, type of cancer, type of treatment and number of children in the family were included in the check list. The literature relating to the effect of childhood cancer on the families was reviewed to inform the development of these factors.

Due to the fact that the current study was conducted in Jordan, all instruments needed to be in Arabic language. The PSS10 instrument has been previously used in Jordan in the Arabic language (Hamdan-Mansour & Dawani, 2008) whereas, the demographic and characteristics check list had not been used in Jordan before. Therefore, the demographic and characteristics check list was translated into Arabic. Special care was taken during the translation process. First of all, the researcher translated the demographic and characteristics check list into Arabic. Secondly, the Arabic version was back translated into English by a professional bilingual translator in Jordan. Finally, both Arabic and English versions were checked by three bilingual people in Jordan and they provided their comments. These comments were taken into consideration and a meeting between all of the translators and the researcher was managed. The check list was discussed and agreement on a specific format was reached.

The researcher checked the applicability of the whole process. The whole process was applied by distributing the invitation letter, information sheet, consent form and the questionnaire. The first ten couples were treated as a pilot to assess feasibility; because there were no changes in the process, the information gained from those couples was included in the analysis and the researcher proceeded with the data collection.

In this study, 405 couples of a child with cancer were invited to participate. Of those, 305 couples signed the consent to participate. Parents who did not complete the questionnaires and/or did not form a couple were excluded from the analysis. At the end, only 300 couples fully completed, returned the questionnaires and were included in this study. Table 1 presents the distributions of the participants and the percentage of the approached/consented couples among the six selected hospitals. ‘Hospital A’ has the highest number of participants; this is expected due to the fact that more than 80% of childhood cancer cases in Jordan are treated there (Jordan National Cancer Registry, 2007). In all of the six hospitals, the percentages of the approached/consented couples were close to each other (i.e. between 69% and 83%) suggesting little recruiting bias. The findings of all of the participant couples are discussed as one group in the paper.

**Table 1:** Distribution of the participants among the selected hospitals

Hospital	Couples approached	Couples consented	Couples were included in the analysis	Percentage of approached/ consented	Percentage of approached/included in the analysis
Hospital A	320	242	240	76%	75%
Hospital B	18	13	12	72%	67%
Hospital C	35	26	25	74%	71%
Hospital D	7	5	4	71%	57%
Hospital E	12	10	10	83%	83%
Hospital F	13	9	9	69%	69%
Total	405	305	300	75%	74%

### 2.6 Data Analysis Process

The Statistical Package for the Social Sciences 17.0 was used to analyse the quantitative data. After coding the data, the researcher entered the survey data into SPSS. The questionnaire data were entered for related couples. The significance level (P-value) of less than 0.05 was considered as statistically significant result and two tailed tests were used in all statistical analysis techniques. Numbers that emerged in the results were rounded up to the closest two decimals points.

Descriptive statistics were computed to describe the study sample and the factors associated with the perceived stress levels. Paired t-tests were the most appropriate statistical technique to investigate the differences between mothers and fathers in their perceived stress scores in term of couple settings. Effect size was calculated for each paired t-test.

An effect size of 0.01 is considered a small effect, 0.06 moderate, and 0.14 is considered a large effect size. These measures are based on Pallant (2007, Page 240).

A one-way between groups analysis of variance (ANOVA) with post-hoc tests was conducted to explore the impact of the child’s diagnosis on the parents. Backward regression analysis was also used to determine the predictors associated with perceived stress in parents of a child with cancer. Dummy variables were created for some predictors in order to include them in the analysis.

Regression was conducted for mothers and for fathers. The same items were included in the mothers’ and fathers’ equations each time. The adjusted R square rather than the R square has been selected when presenting the results as it adjusts for the number of terms in the model. Diagnostics and assumptions appropriate for regression analysis test, as stated by Pallant (2007), were checked (i.e. multicollinearity, outliers, normality, linearity, homoscedasticity and independence of residuals).

## 3. Results

In this study, in order to confirm the reliability of the Arabic version of the PSS10, Cronbach’s alpha coefficient was calculated for mothers and fathers and was 0.89 for mothers and 0.90 for fathers, suggesting very good internal consistency reliability of the scale within this sample.

The respondents (n=300) were couples parenting a child with cancer in Jordan (i.e. 300 mothers and 300 fathers). Fathers were older than mothers in the vast majority (97%) of the couples responding. The range of the age was from 19 to 68 years for fathers and 16 to 52 years for mothers, with a mean of approximately 6 years of age difference within couples.

More than 90% of the parents had high school education and above, with the majority of the responding having a college degree. Findings showed that 90% of the fathers and 11% of the mothers were employed. However, financial status was described as tight by approximately 37% of fathers and 29% of mothers. More than 90% of the families had less than 6 children and 14% of the families had only one child (i.e. the ill child).

**Table 2:** Characteristics of the parents (continuous variables)

Characteristics	Mothers n=300 mean (SD)	Fathers n=300 mean (SD)
Age	34.00 (7.31)	39.99 (8.09)
Total perceived stress score	23.98 (8.21)	20.25 (8.68)
Number of children	2.84 (1.36)	2.84 (1.36)
Age difference between fathers and mothers in years	5.99 (3.38)	

**Table 3:** Characteristics of the parents (non continuous variables)

Characteristics	Mothers n=300 % (n)	Fathers n=300 % (n)
Employed	11.00% (33)	90.00% (270)
Unemployed	89.00% (267)	10.00% (30)
Not educated	1.00% (3)	0.30% (1)
Elementary school	7.00% (21)	9.70% (29)
High school	37.00% (111)	36.30% (109)
College	53.30% (160)	45.70% (137)
University/postgraduate	1.70% (5)	8.00% (24)
Financially comfortable	23.00% (69)	20.30% (61)
Financially varies	47.70% (143)	42.70% (128)
Financially tight	29.30% (88)	37.00% (111)

Mothers had a relatively higher total perceived stress scores than fathers, with a mean of 23.98 (standard deviation 8.21) compared to a mean of 20.25 (standard deviation 8.68) for fathers.

### 3.1 General Characteristics of Children with Cancer

Ages of the ill children ranged between 4 months and 17.60 years, with a median of 6 years. Approximately 57% of the ill children were males and 43% females. Leukaemia was found to be the most common diagnosis, 35% among all children. The children’s diagnosis also included cancer of brain and central nervous system (19%), lymphoma (10%), bone (5%), kidney (4%), eye (7%), connective tissue (2%), nasopharynx (2%), liver (2%), adrenal gland (1%) and testis and other different cancers (14%). This diagnostic classification is aligned with the Jordanian paediatric cancer classification (Jordan National Cancer Registry, 2007). Children had been diagnosed with cancer at different times, with parents filling in the questionnaires a median of 11 months after the diagnosis.

In terms of the type of the treatment that the children had undergone, 36% of the children had surgery, 92% chemotherapy, 14% radiotherapy, 3% bone marrow transplantation and 14% had traditional treatment, with many children receiving more than one type of treatment. The government was responsible for the financial cost of the treatment for 93% of the children and the rest were self funded.

At the time of data collection, children were either hospitalised or were attending outpatient oncology clinics. Almost 65% of all children continue to live at home and attend the paediatric clinics for medication infusions, medical procedures and/or follow-up. The majority of the participants (80% of the sample) were recruited from ‘Hospital A’ (i.e. private sector), 12% from four governmental hospitals and 8% from a military hospital. Three different geographical regions were involved. In this study, 275 couples were recruited from Amman, 12 from Irbid and 13 from Zarqa. Families were living up to 400 KM away from their hospital, with a median of 25 KM.

### 3.2 Differences between Mothers and Fathers

Although most of the parents (i.e. about 46% of the mothers and 60% of the fathers) agreed that caring for an ill child is a shared responsibility between both members of the couple, mothers were the primary caregivers for their ill children in many cases. Approximately 52% of the mothers and 38% of the fathers stated that only mothers were the primary caregivers of their ill child whereas, only 2% of the fathers consider themselves as primary caregivers. In the vast majority of cases, care for the ill child was either provided solely by the mother or jointly by both parents. It was very rare that fathers were the primary caregivers for their ill child. Moreover, mothers and fathers were found to share the decisions related to their ill child, with the majority (i.e. about 90%) considering taking treatment decisions as shared responsibility.

Although the vast majority of the families were not paying for the treatment, having a child with cancer was found to have a financial impact. More fathers than mothers (about 74% and 50%, respectively) found that having a child with cancer was associated with increased expenditure. Losing a job was found to affect mothers more than fathers, as 16% of the mothers lost their job compared to 9% of the fathers. Only 14% of the fathers compared to 28% of the mothers found that having a child with cancer had not affected them financially.

#### 3.2.1 Difference in Perceived Stress Scores between Mothers and Fathers

A paired samples t-test was used to evaluate the difference between mothers and fathers parenting a child with cancer in their total perceived stress scores. Findings showed that there was a significant difference in perceived stress scores between mothers and fathers parenting a child with cancer (p<0.001), with mothers reporting significantly higher stress scores (i.e. 23.98 compared to 20.25). The mean difference between them was 3.73, (95% CI: 3.08 to 4.36) and the eta squared statistic=0.30 indicated a large effect size.

A one-way analysis of variance was conducted to explore the impact of the child’s diagnosis on the mothers’ stress levels. Subjects were divided into four groups according to their child’s diagnosis (i.e. Leukaemia, brain and CNS, lymphoma and other types of childhood cancer). There was a statistically significant difference in the mothers stress levels between the four groups: f (3, 296)=6.84, p<0.001. Eta squared=0.06 indicated a moderate effect size. Post-Hoc comparisons tests indicated that the mean score for lymphoma was significantly lower than all other diagnostic groups but there was no statistical difference in the maternal stress levels between the other diagnostic groups.

**Table 4:** The mean stress scores for mothers across different cancer diagnoses

Child’s diagnosis	Mothers n=300	Mean of maternal stress scores	SD
Leukaemia	104	24.70	8.28
Brain and CNS	56	25.64	7.00
Lymphoma	30	17.93	5.96
Others	110	24.09	8.60

**Table 5:** Post-Hoc tests of total maternal stress scores across different cancer diagnoses

Diagnosis		Mean difference in the maternal stress levels	P value
Leukaemia	Brain and CNS	-0.94	0.89
	Lymphoma	6.77	<0.001
	Others	0.61	0.94
Brain and CNS	Leukaemia	0.94	0.89
	Lymphoma	7.71	<0.001
	Others	1.55	0.64
Lymphoma	Leukaemia	-6.77	<0.001
	Brain and CNS	-7.71	<0.001
	Others	-6.16	<0.01
Others	Leukaemia	-0.61	0.94
	Brain and CNS	-1.55	0.64
	Lymphoma	6.16	<0.01

A one-way analysis of variance was conducted in order to examine whether there are any significant differences in the mean scores of paternal stress levels across different diagnoses in their children. As for the maternal analysis, participants were divided into four groups according to their child’s diagnosis (i.e. Leukaemia, brain and CNS, lymphoma and other types of childhood cancer). There was a statistically significant difference in the fathers stress levels between the four groups: f (3, 296)=5.69, p<0.01. Eta squared=0.05 indicated a moderate effect size. Post-Hoc comparisons tests indicated that the mean score for lymphoma was significantly lower than all other diagnostic groups but with no statistical difference in the paternal stress levels between the other diagnostic groups.

**Table 6:** The mean stress scores for fathers across different cancer diagnoses

Child’s diagnosis	Fathers n=300	Mean of paternal stress scores	SD
Leukaemia	104	20.53	9.11
Brain and CNS	56	22.00	8.13
Lymphoma	30	14.40	7.01
Others	110	20.70	8.40

**Table 7:** Post-Hoc tests of total paternal stress scores across different cancer diagnoses

Diagnosis		Mean difference in the paternal stress levels	P value
Leukaemia	Brain and CNS	-1.47	0.72
	Lymphoma	6.13	<0.01
	Others	-0.17	0.99
Brain and CNS	Leukaemia	1.47	0.72
	Lymphoma	7.60	<0.01
	Others	1.30	0.79
Lymphoma	Leukaemia	-6.13	<0.01
	Brain and CNS	-7.60	<0.01
	Others	-6.30	<0.01
Others	Leukaemia	0.17	0.99
	Brain and CNS	-1.30	0.79
	Lymphoma	6.30	<0.01

### 3.3 Predictors of High Stress Levels for Mothers/Fathers of a Child with Cancer in Jordan

Factors associated with stress were identified in the literature review and where available were used in the regression equations. Those child and family characteristics hypothesised to be associated with the mother’s/father’s stress level were entered: Age of the mother/father, job status of the mother/father (employed/unemployed), number of children in the family, paying for treatment (self-funded/insurance), time since diagnosis of the ill child, child’s location (admission/outpatient), child’s diagnosis, age of the ill child, gender of the ill child, distance to the hospital and partner’s stress score.

#### 3.3.1 Predictors of High Stress Levels for Mothers

The factors that remained significant at the end of equation are presented in Table 8.

**Table 8:** Predictors accepted to be in the mother’s model

Variables accepted in the model	Standardised Beta	T	P value	Part
Number of children	-0.10	-2.87	<0.01	-0.10
Time since diagnosis	-0.08	-2.08	0.04	-0.07
Child’s location	-0.09	-2.26	0.03	-0.08
Distance to hospital	-0.09	-2.67	<0.01	-0.09
Total paternal stress score	0.72	18.58	<0.001	0.65

*(Variables removed in order: child’s diagnosis, age of the ill child, age of the mother, paying for treatment, gender of the ill child and job status of the mother)*.

3.3.1.1 Model Findings

The final model had an adjusted R square of 0.64, indicating that 64% of the variance in the total maternal perceived stress score is explained by the final model (which includes the variables of number of children, time since diagnosis, child’s location, distance to hospital and total paternal stress score) and the model is significant (p<0.001).

Table 8 shows that the largest standardised absolute Beta value is 0.72, which is for total paternal stress score indicating that this variable makes the strongest unique contribution to explaining the maternal stress score (i.e. an increase of fathers’ stress scores predicts an increase in the maternal stress scores). A decrease in number of children in the family, time since diagnosis and distance to hospital predict an increase in the mothers’ stress scores. Moreover, when the child is an inpatient this predicts an increase in the mothers stress scores.

#### 3.3.2 Predictors of High Stress Levels for Fathers

The factors that remained significant at the end of equation are presented in Table 9.

**Table 9:** Predictors accepted to be in the father’s model

Variables accepted in the model	Standardised Beta	T	P value	Part
Father’s age in years	-0.18	-4.50	<0.001	-0.15
Job status of the father	0.14	4.02	<0.001	0.14
Number of children	0.09	2.48	0.01	0.09
Paying for treatment	-0.09	-2.55	0.01	-0.09
Distance to hospital	0.08	2.43	0.02	0.08
Total maternal stress score	0.73	19.66	<0.001	0.67

*(Variables removed in order: child’s diagnosis, time since diagnosis, child’s location, age of the ill child and gender of the ill child)*.

3.3.2.1 Model Findings

The final model had an adjusted R square of 0.65 indicating that 65% of the variance in the total paternal perceived stress score is explained by the final model (which includes the variables of age of the father, job status of the father, number of children, paying for treatment, distance to hospital and total maternal stress score) and the model is significant (p<0.001).

Table 9 shows that the largest standardised Beta value is 0.73, which is for total maternal stress score indicating that this variable makes the strongest unique contribution to explaining the paternal stress score (i.e. an increase of the mothers’ stress scores predicts an increase in the paternal stress scores). An increase in the distance to hospital, number of children in the family, father being unemployed and parents paying for medical expenses were all predictors associated with an increase of fathers’ stress scores whereas, a decrease in the age of the fathers predicts an increase in their perceived stress scores.

Proposed Research Model for Mothers and for Fathers

**Figure 1:**
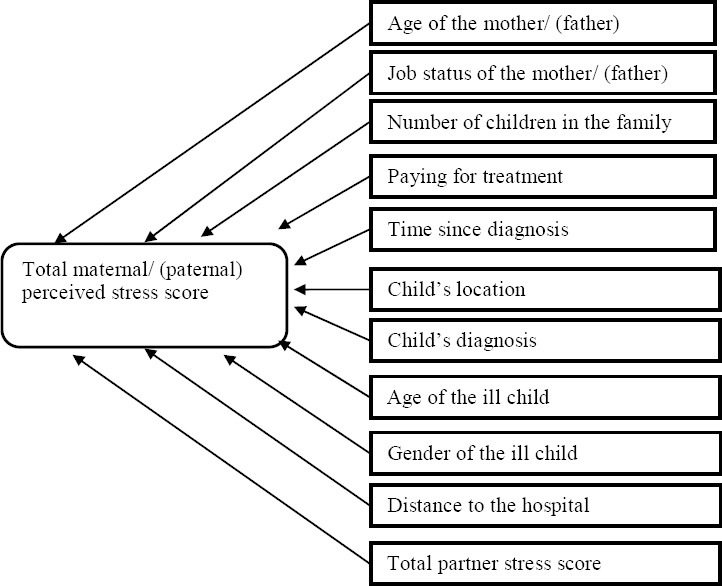
Proposed parental stress model

The final regression model for mothers

**Figure 2:**
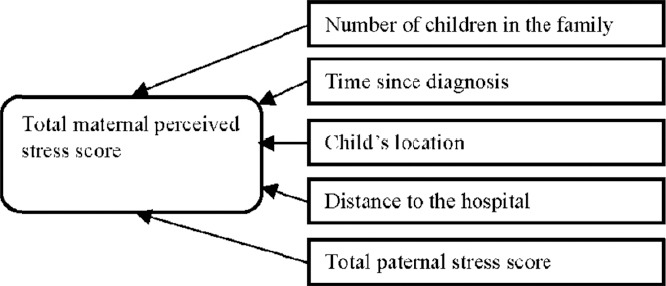
Predictors of maternal stress

The final regression model for fathers

**Figure 3:**
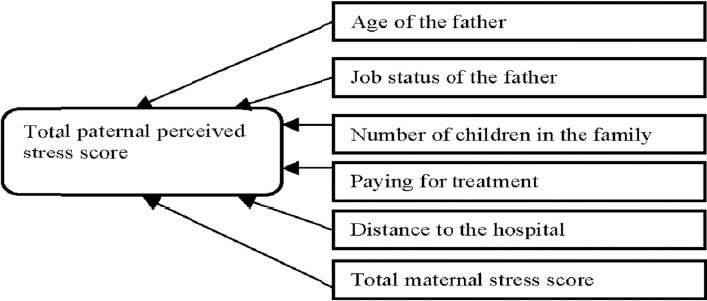
Predictors of paternal stress

## 4. Discussion

Results showed that when a child has cancer, the proportion of mothers who work is significantly lower than the proportion of fathers who work (i.e. 11% of mothers were employed compared to 90% of the fathers). This is a lower percentage than the wider Jordanian population where 26% of mothers of school aged children were in paid employment (Masa’deh, Collier, & Hall, 2012). This is in agreement with international studies from China and Canada which both reported that very few fathers of children with cancer compared to mothers had left their job after their child have been diagnosed with cancer (Fletcher, 2010; Limburg et al., 2008; Yeh, 2002). In the current study, this is likely to be because mothers were the primary caregivers for their ill child in the vast majority of the sample.

Mothers of a child with cancer reported significantly higher stress scores than fathers. A direct explanation of why mothers reported higher stress scores than fathers is that mothers, in the majority of the participants, were the primary caregivers to their ill child. This means the mothers were physically closer to their ill child than fathers and were the ones who interacted with their ill child most of time and dealing directly with the effects of treatment. This finding is in agreement with previous studies investigating stress in parents of children with cancer and which showed that mothers typically reported higher stress scores than fathers (Bayat et al., 2008; Norberg, Lindblad, & Boman, 2005; Norberg et al., 2006; Patino-Fernandez et al., 2008; Pöder et al., 2008; Pöder, Ljungman, & von Essen, 2010; P. Sloper, 1996; Wijnberg-Williams, Kamps, Klip, & Hoekstra-Weebers, 2006; Yeh, 2002). Just one study reported no differences between mothers and fathers of a child with cancer in their reported quality of life (Goldbeck, 2001). However, this study acknowledged that there were some differences between mothers and fathers in their perceived demands and resources (i.e. mothers had more support resources than fathers). As the sample in Goldbeck’s study was very small (i.e. 25 German couples) caution is required in the generalisation of the results.

Findings in the current study showed that both mothers and fathers had significantly higher stress scores than mothers and fathers in general population (Masa’deh et al., 2012). Mothers had significantly higher stress levels compared to fathers, with a large effect size and is in line with previous research suggests that mothers in general report higher stress levels than fathers (Clarke, McCarthy, Downie, Ashley, & Anderson, 2009; Masa’deh et al., 2012; Yeh, 2002). Lazarus (2006) identified that stress happens when the perceived demands of an individual exceed the perceived resources. The last hypothesis is likely to reflect the situation in this study, particularly, because the factors associated with high stress levels were different between mothers and fathers.

### 4.1 Discussion of the Regression Models

#### 4.1.1 Age of the Parent and Perceived Stress Levels

In the current study, the age of the parent was found to be negatively associated with stress levels in fathers (younger fathers reporting higher stress scores than older ones). This is aligned with many previous studies which have found that perceived stress in general has a negative correlation with age and younger participants report higher stress levels than older participants (Cohen & Williamson, 1998; Remor, 2006). Remor (2006) used the European Spanish version of PSS10 to measure stress levels for males and females from different groups of Spanish people between 18-69 years old, with an average of 31.70 years old. Result showed that reported stress was affected by age of the participants. Age was significantly inversely correlated with the PSS10, suggesting that perceived stress tend to decline as age increases. Earlier, Cohen and Williamson (1998) used the PSS10 to measure stress in the normal population in the USA. Age of the participants was classified into five categories. Results showed that the PSS10 correlated negatively with age (the younger group had the higher PSS10). Additionally, Aldwin et al. (1996) found that although older people expended less coping effort than younger people, they reported less stress than young people group. This might provide at least part of the explanation of why age was a significant factor in the final models in this study.

On the other hand, one study conducted in the UK measured stress for 289 participants between 18-50 years of age from four health professionals and showed no relationship between stress and age (Birks, McKendree, & Watt, 2009). However, this study included a particular group of people (i.e. only professionals) and therefore the results cannot be broadly generalised to the general population.

#### 4.1.2 Employment Status of the Parent and Perceived Stress Levels

In this study, the job status of the parent (employed vs. unemployed) was found to be predictor of stress levels for the fathers but not the mothers. Although more women than men reported that they left their job to stay with the ill child, fathers’ but not mothers’ high stress levels were associated with being unemployed. Many previous studies showed that more mothers left their job than fathers in order to care for an ill child (Ångström-Brännström, Norberg, Strandberg, Söderberg, & Dahlqvist, 2010; Fletcher, 2010; Gravestock et al., 2011; Lähteenmäki, Sjöblom, Korhonen, & Salmi, 2004; Limburg et al., 2008; Sloper, 1996; Yeh, 2002). For example, Yeh (2002) studied parents of a child with cancer in Taiwan and demonstrated that although approximately 35% of mothers were working before their child was diagnosed with cancer, all those mothers stopped working to stay with their ill child during hospitalisation. Moreover, parents of a child with cancer in the United Kingdom have reported that their employment was negatively affected by their child’s illness with one study showing that at least one of the parents had to resign in order to care for the ill child (Sloper, 1996). Studies showed that high anxiety and depression levels of parents caring for children with cancer are associated with parents losing their jobs as they are the primary caregivers for the ill child (Hoekstra-Weebers, Jaspers, Kamps, & Klip, 2001; Rajajee et al., 2007; Sloper, 2000). However, the vast majority of the primary caregivers considered in these studies were mothers.

Cultural differences may contribute to the results. In the general Jordanian population, approximately 75% of mothers are housewives (Jordanian Department of Statistics, 2007). Therefore, being unemployed in Jordan may be viewed as being quite acceptable for the mothers but not for the fathers. This might contribute to one possible explanation of why being unemployed affected only the father’s stress levels despite the fact that the vast majority of the fathers of a child with cancer were working compared to only 11% of the mothers.

#### 4.1.3 Paying for Treatment and Perceived Stress Levels

Cancer treatment in general is very expensive. Families who care for a child with cancer face financial costs during the diagnostic, treatment, and follow-up care phases of the disease (Dockerty et al., 2003; Eiser & Upton, 2007; Fletcher, 2010; Limburg et al., 2008). The high cost of cancer treatment, particularly for those with no medical insurance cover, combined with other expenses have been found to be associated with high stress levels in parents caring for a child with cancer (Dockerty et al., 2003; Gravestock et al., 2011; Miedema et al., 2008).

In the current study, the cost of treatment for the children with cancer was either paid by the family or by a third party (i.e. insurance companies or governmental charities) and more than 90% of the families stated that the treatment cost of their ill child was paid by a third party. In the final regression models, high stress levels of fathers were found to be associated with those who were not financially covered and were paying for the treatment costs themselves. This finding is consistent with previous studies stating that childhood cancer has an effect on the family financial status and therefore on their perceived stress levels (Dockerty et al., 2003; Gravestock et al., 2011; Limburg et al., 2008; Miedema et al., 2008). For example, a cross-sectional survey of 192 families caring for children who were diagnosed with different types of cancer in New Zealand where people receive cancer treatment free of charge showed that approximately 89% of those families have received some compensation for expenses from different resources. Of the families, 37% reported that they needed to borrow money because of the financial effects of their child’s illness (Dockerty et al., 2003). Consequently, those who pay the cost of the treatment themselves, which may be a tremendous amount of money, are expected to be more financially affected and in turn have higher stress levels than those whose treatment costs are covered by a third party.

#### 4.1.4 Time since Diagnosis and Perceived Stress Levels

Findings showed that time since diagnosis was a predictor of stress levels for mothers. A shorter time since the child was diagnosed with cancer was associated with higher stress levels for both parents. Many previous studies have investigated perceived stress in parents of children with cancer over time and found that parents have the highest stress levels at the time of diagnosis of their children with cancer (Chesler & Parry, 2001; Goldbeck, 2006; Han, 2003; Maurice-Stam et al., 2008; Nicholas et al., 2009; Patistea et al., 2000; Wong & Chan, 2006). Patino-Fernandez et al. (2008) investigated stress in parents (128 mothers and 72 fathers) of children newly diagnosed with different types of cancer in the United States. Parents completed various stress questionnaires and acute stress symptoms were found to be significant in about 50% of them two weeks after the diagnosis. The authors of the study suggested a psychiatric consultation for all parents of children with cancer at the time of diagnosis.

In the current study, one possible explanation of why a shorter time since the child was diagnosed with cancer predicts high perceived stress levels in the final mother’s models is that mothers might not have enough information in the beginning about their child illness. Moreover, previous studies showed that at the time of diagnosis parents reported high stress levels caused by disbelief, facing the risk of mortality, having fundamental feelings of security disappear and falling into a state of the unknown because they do not have sufficient information about their child’s illness and causes of the disease (Bjork et al., 2005; Maurice-Stam et al., 2008; Wong & Chan, 2006).

Stress associated with a cancer diagnosis has been found to decrease with time. Perceived stress was measured three times in a longitudinal study of women with breast cancer, with approximately a year difference between each time. The results showed that perceived stress decreased significantly over time (Golden-Kreutz, Browne, Frierson, & Andersen, 2004). The authors suggest that positive coping might be the reason for the difference in perceived stress levels. It is possible that such coping might explain why stress decreased over time since diagnosis in the current study. However, stress has been investigated for 126 parents in the United Kingdom six months (i.e. time one) and eighteen months (i.e. time two) after their child was diagnosed with different types of cancer. This study found that although parents reported high levels of stress at time of diagnosis, these levels did not change significantly over time (Patricia Sloper, 2000). This study did not take the expected survival into account, approximately 15% of those participated in time one lost their child after that and did not participate in time two; this might have a significant effect on the results.

Even though a few studies report that parental stress does not significantly change over time, the majority of the previous studies found that such stress decreased with time. Pai et al. (2007) reviewed 29 articles published between 1967 and 2005 which examined the effect of paediatric cancer on parents. Their review concluded that the first year after diagnosis was the most stressful period for the parents. However, such stresses often decrease over time and the findings in the current study are in agreement.

#### 4.1.5 Child In/Outpatient and Perceived Stress Levels

Findings that emerged from this study indicated that high perceived stress levels for mothers were associated with the child being in hospital. Children with cancer are normally admitted to hospital either to receive some aggressive treatment, or because the child’s health status may need some careful and professional observation (Colletti et al., 2008; Eiser, 2004; Whitsett, Gudmundsdottir, Davies, McCarthy, & Friedman, 2008). In addition to that, the whole family life may change when a child stays in hospital (McGrath & Phillips, 2008; Yeh, 2002). One parent often stays with the ill child and the other needs to manage the normal daily activities and care for other children.

The medical status of the ill child and the demanding life situation when the ill child is in hospital may explain why being in hospital was associated with high perceived stress levels in parents of a child with cancer in this study. This is in agreement with previous studies exploring the effect of treatment of childhood cancer on parents. Previous studies found that parents perceive the time of the treatment of their children as stressful, and this period of time is associated with different medical investigation, procedures, appointments and schedules (McGrath, 2001; McGrath & Phillips, 2008). Hospitalisation, treatments and common possible side-effects were considered a cause of stress for the ill child and the parents (McGrath, 2001). Increased parental stress levels whilst the child is in hospital are aligned with two different studies that measured stress levels in parents of a child with cancer receiving treatment and compared these with parents of a child who had completed their cancer treatment. Parents of children receiving treatment showed higher stress levels than the ‘completed treatment’ group (Kazak et al., 2005; Norberg et al., 2005).

Further information from some previous studies showed that outpatient treatment settings might also include some stressors for the parents. Parents are likely to experience time pressure as having a child with cancer needs constant care and attention. The parents must often learn to manage complex medications and monitoring the child’s general health status at home (James et al., 2002; McGrath & Phillips, 2008; Woodgate & Degner, 2003). However, these studies still acknowledged that being in hospital is more demanding for the parents.

#### 4.1.6 The Effect of the Number of Children in the Family and Distance to Hospital on Perceived Stress Levels

Interestingly, the number of children in the family and distance to hospital were found to affect mothers and fathers in different ways. A decrease in the number of children and distance to hospital predicted high stress levels for mothers whereas an increase in the number of children in the family and distance to hospital predicted high stress levels for fathers parenting a child with cancer in Jordan. In this study, the vast majority of the mothers were the primary caregivers for their ill child. It was very rare that fathers were the primary caregivers for their ill child. Therefore, fathers were often expected to take care of the other children. Although some families stated that grandparents helped them in caring for other healthy children, approximately 32% of the mothers and fathers reported that no-one helped them in caring for other children. In most of the cases, both parents had role changes once their child was diagnosed with cancer. This is consistent with a previous study which showed that once the mother takes on the role of primary caregiver for the ill child (particularly, if the ill child is in the hospital) the father’s role in the family changes, taking on the majority of what was previously the mother’s work (Chesler & Parry, 2001). Fathers of a child with cancer in the United States were found to be responsible for some of the household duties and to help with the care for the ill child and other children, besides keeping their original parental roles (Chesler & Parry, 2001). Accordingly, for fathers of a child with cancer, an increase in the number of children in the family often means increase in their new responsibilities as a household and a carer for the healthy children.

Fathers were found to be more stressed when there was greater distance to hospital. On the one hand, fathers were possibly visiting their ill child in the hospital. This means more effort, time and money consuming for the fathers. Additionally, mothers who stay with the ill child in the hospital may go home less often if they live away from to the hospital and this may affect the paternal roles within the family. On the other hand, fathers might also be concerned about distance when their ill child was being cared for at home. In this case, fathers thought about the consequences of any emergency situation happen to their ill child; this might mean time consuming and could be life-threatening at times as well. This could be an explanation of why longer distance is associated with higher stress levels for fathers.

#### 4.1.7 The Effect of the Partner Stress Score on Perceived Stress Levels

High perceived stress levels for a parent in this study were found to be associated with high perceived stress levels in their partners. The higher mothers’ stress scores were associated with higher fathers’ stress scores and vice versa. Due to the fact that mothers and fathers in this study were couples sharing almost the same life situation, economical status, demographics and mainly the same ill child situation, mothers’ and fathers’ stress levels could be expected to be high or low together at the same time and would explain why partner’s stress score was a predictor in the models. Although mothers’ stress scores were found to be higher than fathers, increased stress levels for mothers were associated with increased stress levels for fathers.

Stress researchers traditionally have focused on stressful life events that people experience personally and individually, ignoring the potentially disruptive effects of stressful events experienced by family members. The results that emerged from this study showed that mothers and fathers stress is affected by their partners stress levels. This is consistent with Westman (2001) and Kim et al. (2010) who identified that stress might be seen as a dynamic of a unit and not only as an individual. Additionally, researchers believed that not only your own stress level can affect your health but also your spouse stress level can affect you as well (Youngmee et al., 2010; Youngmee et al., 2008). However, participants in Kim’s studies were couples where one of them had cancer. Other researchers investigated the crossover of stress between partners and Westman et al. (2001) and Bakker et al. (2005) found that stress could be transmitted from husband to wives and vice versa, affecting their psychological health.

With regards to parents of a child with cancer, results that have emerged from the current study indicate that there is a huge effect of the spousal stress on their partners as hypothesised by Stuber (1995) who suggested that there may be a chance that stress levels of mothers and fathers of a child with cancer could also be affected by each other. However, Stuber (1995) acknowledged that this interaction may be complicated and vary between families; as many couples may share the child care responsibilities, care of others children and normal life issues. Yet, this research is the first conclusive evidence that spousal stress levels affect partners when having a child with cancer.

## 5. Conclusions

Consistent with previous studies that investigated perceived stress in parents of children with cancer, the results showed that mothers reported significantly higher perceived stress scores than fathers. There was an effect of the type of cancer diagnosis on the parental stress levels for both parents. Mothers and fathers reported different factors associated with their stress scores. However, the partner’s stress score made the largest contribution to the prediction of parental stress. The findings provide evidence of the need for psychological support to be developed for families caring for a child with cancer in Jordan. Whilst Middle Eastern countries have many similarities in their culture and religion, the findings of this research may also benefit parents of children with cancer in other surrounding countries.
